# Cell cycle times of short-term cultures of brain cancers as predictors of survival

**DOI:** 10.1038/sj.bjc.6604716

**Published:** 2008-10-14

**Authors:** C E Furneaux, E S Marshall, K Yeoh, S J Monteith, P J Mews, C A Sansur, R J Oskouian, K J Sharples, B C Baguley

**Affiliations:** 1 Department of Neurosurgery, Auckland Hospital, Auckland, New Zealand; 2 Auckland Cancer Society Research Centre, University of Auckland, Auckland, New Zealand; 3 Department of Preventive and Social Medicine, University of Otago, Dunedin, New Zealand

**Keywords:** paclitaxel, glioblastoma, melanoma, primary culture, T_pot_

## Abstract

Tumour cytokinetics estimated *in vivo* as potential doubling times (*T*_pot_ values) have been found to range in a variety of human cancers from 2 days to several weeks and are often related to clinical outcome. We have previously developed a method to estimate culture cycle times of short-term cultures of surgical material for several tumour types and found, surprisingly, that their range was similar to that reported for *T*_pot_ values. As *T*_pot_ is recognised as important prognostic variable in cancer, we wished to determine whether culture cycle times had clinical significance. Brain tumour material obtained at surgery from 70 patients with glioblastoma, medulloblastoma, astrocytoma, oligodendroglioma and metastatic melanoma was cultured for 7 days on 96-well plates, coated with agarose to prevent proliferation of fibroblasts. Culture cycle times were estimated from relative ^3^H-thymidine incorporation in the presence and absence of cell division. Patients were divided into two groups on the basis of culture cycle times of ⩽10 days and >10 days and patient survival was compared. For patients with brain cancers of all types, median survival for the ⩽10-day and >10-day groups were 5.1 and 12.5 months, respectively (*P*=0.0009). For 42 patients with glioblastoma, the corresponding values were 6.5 and 9.0 months, respectively (*P*=0.03). Lower grade gliomas had longer median culture cycle times (16 days) than those of medulloblastomas (9.9 days), glioblastomas (9.8 days) or melanomas (6.7 days). We conclude that culture cycle times determined using short-term cultures of surgical material from brain tumours correlate with patient survival. Tumour cells thus appear to preserve important cytokinetic characteristics when transferred to culture.

*In vivo* tumour proliferation rates, initially estimated from the percentages of either mitotic cells or S-phase cells, have long been known to be related at least for some tumour types to clinical outcome (Tannock, 1978). Other estimates of proliferative activity, including staining with antibodies to antigens such as Ki-67 and PCNA, and more recently, gene expression profiles (Nutt *et al*, 2003), have also been associated with clinical outcome in some studies. Potential tumour doubling times (*T*_pot_ values) rely on simultaneous measurement of S-phase percentage and S-phase duration and provide more quantitative estimates, which for many tumour types cover a range from 2 days to several weeks (Wilson *et al*, 1988; Rew and Wilson, 2000). However, although short *T*_pot_ values are generally related to poor prognosis, the value of *T*_pot_ as an independent prognostic indicator is controversial (Haustermans and Fowler, 2001), and in the case of brain cancers, *T*_pot_ values are not clearly related to survival (Danova *et al*, 1988; Struikmans *et al*, 1998).

We have previously investigated the possibility that data from short-term cultures of clinical tumour material might have prognostic significance (Marshall *et al*, 1992, 2003; Baguley *et al*, 2002), and we were particularly interested in the possibility of using tumour material to estimate culture cycle time. Direct measurement in such cultures is impossible because of the presence of host cells in the sample and the loss of tumour cells, through apoptosis or other pathways, during primary culture. However, we had previously found using a series of human tumour cell lines that the degree of incorporation of ^3^H-thymidine into DNA at different times after addition of paclitaxel, an inhibitor of mitosis and cell division, was a function of the measured culture doubling time (Baguley *et al*, 1995). We assumed that culture-doubling time was similar to culture cycle time for cell lines (i.e., that cell loss was negligible) and developed an empirical formula that related culture cycle time to the ^3^H-thymidine data.

We then considered whether the above formula, obtained using cell lines, might be applied to short-term (7-day) cultures of tumour samples obtained at surgery. The nature of the assay (comparison of ^3^H-thymidine incorporation with and without paclitaxel) minimised the consequences of tumour cell loss during culture because any cell loss would affect cultures with and without paclitaxel almost equally. Surprisingly, culture cycle times estimated in this manner were found to vary among individual samples from approximately 2 days to more than 40 days, a range that was remarkably similar to that obtained in *T*_pot_ measurements (Baguley and Marshall, 2004). Furthermore, in a small study of 16 patients with ovarian cancer, derived culture cycle times were found to be related to patient survival (Baguley and Marshall, 2004). Here, we have addressed the question of whether the same approach might be applied to patients with brain tumours. We cultured samples from a range of brain tumours and have also included melanomas metastatic to the brain, as they are also tumours derived from the neural crest. We then determined whether culture cycle times derived by this approach were related to overall survival.

## Materials and methods

### Culture of tumour samples

Patients received surgery in the Department of Neurosurgery, Auckland City Hospital. All studies were carried under guidelines approved by the Northern X Regional Ethics Committee, and informed consent was obtained from all patients. A portion of tumour tissue taken from patients undergoing surgery for brain malignancies was placed in *α*-MEM growth medium containing insulin (10 *μ*g/ml), transferrin (10 *μ*g/ml), selenite (10 ng/ml) and 5% fetal bovine serum and was used either immediately or after overnight storage at 4°C. Tumour material was disaggregated to form small cellular clusters to preserve, as far as possible, cell–cell and cell–matrix interactions (Baguley *et al*, 2002). Preparations were monitored by phase contrast microscopy and by examination of haematoxylin/eosin-stained cytospins. Cell suspensions, containing both single cells and cell clusters, were transferred to 96-well tissue culture plates that had previously been coated with a thin layer of agarose to prevent the growth of fibroblasts and were grown at 37°C under an atmosphere of 5% O_2_, 5% CO_2_ and 90% N_2_ (Marshall *et al*, 1992).

### Determination of culture cycle times

**Figure 1:**
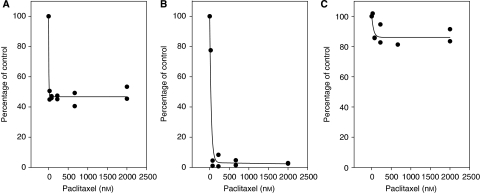
Examples of ^3^H-thymidine incorporation as a function of paclitaxel concentration. Profiles are shown for patients D4 (**A**), S4 (**B**) and B6 (**C**), with designations in Table 1.

Plates were set up to contain between 940 and 7500 cells in a volume of 150 *μ*l, to allow selection of a cell density where ^3^H-thymidine incorporation after 7 days was proportional to the initial number of cells added. Cultures were grown either in the absence of drug or in the presence of paclitaxel at five 3-fold concentration steps up to a maximum of 2 *μ*M. S-phase cells remaining at the end of the incubation were labelled by the addition of ^3^H-thymidine over the last 24 h (Baguley *et al*, 1995). Cultures were harvested and duplicate samples were analysed for each paclitaxel dose with multiple control samples. ^3^H-thymidine incorporation data in the presence of paclitaxel were fitted by a least-squares fit to an exponential of the form *y*=*P*+*a*e^−*b*x^, where *y* is the radioactivity (corrected for background), *x* is the paclitaxel concentration and *a* and *b* are variables, as shown in the two examples in Figure 1. Values of *P* therefore reflected the proportion of remaining S-phase cells at the end of the incubation period, which in turn reflected the proportion of G_1_-phase cells that were feeding into S-phase. The estimated culture cycle time in days (*T*) was calculated using an equation *T*=−3.78/(log_10_ *P*), which was derived from empirical data obtained with cell lines (Baguley *et al*, 1995). When *P* was >0.81 (*T*>40), culture cycle time was not accurately provided by this formula and was arbitrarily set at 40 days.

### Statistics

Median culture cycle times were compared using the Kruskall–Wallis test, and proportions using a *χ*^2^ test. Survival was compared using Kaplan–Meier survival curves with log rank tests and Cox regression. Analysis was carried out using Stata version 9.

## Results

Incorporation of ^3^H-thymidine by primary cultures in the presence of paclitaxel were found to decrease with increasing drug concentration to a ‘plateau’ value (Figure 1) from which the culture cycle time was calculated. Culture cycle times estimated using this method ranged from 2 to ⩾40 days, with a median of 10 days (Table 1). Survival times for the patients from whom tumour samples were taken, together with data for age, gender, tumour grade and treatment, are also shown in Table 1.

Table 2 compares survival and culture cycle times for the different age, gender, tumour type and treatment groups. Survival varied by age (*P*=0.002) and the estimated median survival times in months were 9.5 for those aged under 30 years, 30.7 for those aged 30–50 years, 9.1 for those aged 50–59 years, 7.3 for those aged 60–69 years and 2.6 for those aged 70 years and above. There was no difference in survival by gender (the median survival times were 8.9 months for females and 8.2 months for males, *P*=0.9). Survival was also related to tumour type (*P*=0.0004, Table 2). For patients with glioblastoma, the proportion alive after 1 year was estimated to be 28%, compared with 64% for patients with astrocytoma/oligodendroglioma, 67% for patients with medulloblastoma and 31% for patients with metastatic melanoma. Some of the patients received radiotherapy (median dose 56 Gy), but data were not available for all patients. The median survival times for patients receiving radiotherapy and those not receiving radiotherapy were 12.1 and 2.6 months, respectively (*P*=0.0004). Only nine patients received chemotherapy, most of whom also received radiotherapy.

There was no evidence of variation in culture cycle times by age or gender (Table 2), but patients with astrocytomas or oligodendrogliomas had longer culture cycle times (the percentage with culture cycle times of ⩽10 days was 15%, as compared with 53% for glioblastomas, 50% for medulloblastomas and 75% for metastatic melanomas, *P*=0.04). Patients who had received radiotherapy also had longer culture cycle times (the percentage with culture cycle times ⩽10 days was 35%, compared with 58% for those who did not receive radiotherapy and 78% for those where treatment status was unknown, *P*=0.04).

**Figure 2:**
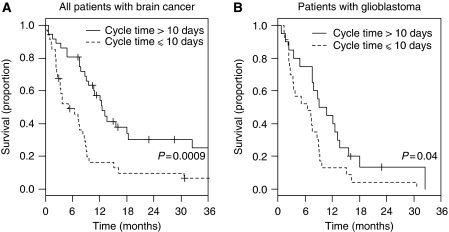
Kaplan–Meier survival plots of patients whose culture cycle times were ⩽10 days (dashed line) and >10 days (solid line). (**A**) All patients. (**B**) Patients with glioblastoma.

Patients were divided into two groups with culture cycle times of ⩽10 days and >10 days. Kaplan–Meier survival curves are plotted in Figure 2A. The median survival times were 5.1 months (95% CI (2.6–8.2)) and 12.5 months (95% CI (9.0–18.4)) for those with culture cycle times of ⩽10 days and >10 days, respectively (*P*=0.0009). Those with culture cycle times ⩽10 days had a 2.4-fold increased risk of death compared with those whose culture cycle times were >10 days (95% CI (1.4–4.2), *P*=0.001). Adjustment for age and tumour type did not explain the differences (Table 3), and adjustment for radiotherapy increased the hazard ratio slightly, giving an estimated 2.9-fold increase in risk among those with shorter culture cycle times (95% CI (1.5, 5.9), *P*=0.003), although this estimate may be biased due to missing data on radiotherapy.

For the 43 patients with glioblastoma, survival (Figure 2B) was also shorter in those with culture cycle times ⩽10 days (*P*=0.04). The median survival times were 6.5 months (95% CI (2.6–8.5)) for those with culture cycle times ⩽10 days and 9.0 (95% CI (4.7–13.7)) for those with culture cycle times >10 days. Those with culture cycle times ⩽10 days had a 2-fold increased risk of death compared with those with culture cycle times >10 days. Survival was also associated with age in this subgroup (*P*=0.05), but culture cycle time was not related to age. For the other tumour types, numbers were generally too small for meaningful comparisons of survival, but it was noteworthy that lower-grade gliomas had a longer median culture cycle time (16.0 days) and were associated with a longer median survival (66.4 months) than the other tumour types.

## Discussion

The results show a significant relationship between survival and culture cycle times derived from short-term cultures of tumour samples taken at surgery from 70 patients with brain cancer. Culture cycle times varied from 2 days to more than 40 days, and the analysis method chosen was to divide patients into two approximately equal groups with culture cycle times of ⩽10 days and >10 days and to compare Kaplan–Meier survival curves fore each (Figure 2). These provided median survival times of 5.1 and 12.5 months, respectively, with a significant survival difference (*P*=0.0009). The study also demonstrated that culture cycle time was independent of age and tumour type. The results can be compared with those in a previous study of 16 patients with ovarian cancer treated with carboplatin, where long culture cycle times was associated with increased complete remission rate (Marshall *et al*, 2002; Baguley and Marshall, 2004). Re-analysis of this data using the methods employed here showed a median survival time for the ⩽10-day group of 2.9 months (95% CI (0.1–11.8)), which was significantly shorter (*P*=0.0003) than the median survival time for the >10-day group of 25.6 months (95% CI (11.8–100)). We also have evidence for similar relationships in a larger group of ovarian cancer patients and in a group of patients with melanoma (unpublished results). These studies are the first to our knowledge to identify *in vitro* proliferation rates of primary tumour cell cultures as a potential marker for survival.

The method used here to estimate culture cycle time diverges significantly from the *T*_pot_ (potential doubling time) method that has been employed extensively in the past as a cytokinetic parameter (Wilson *et al*, 1988; Rew and Wilson, 2000) and deserves comment. The stathmokinetic approach employed relies on the principle that if cell cycle progression is blocked in a certain phase, the proportion of cells in that phase will increase, whereas the proportions in other phases decrease, and that the rate of change of these proportions are a function of culture cycle time. In the specific case of a mitotic poison such as paclitaxel, the number of S-phase cells in control cultures will increase exponentially with time, whereas the number of S-phase cells in drug-treated cultures will decrease with time as a consequence of depletion of the pool of G_1_-phase cells. The concentration of paclitaxel must be high enough to prevent all cell division, accounting for the shape of the dose–response curves in Figure 1. There are two methods of deriving a relationship between ‘plateau’ values (*P*) for ^3^H-thymidine incorporation in these dose–response curves and culture cycle time (*T*). The first, employed here, is to use an empirical formula *T*=−3.78/(log_10_ *P*) established from a series of cell lines and making the assumption, which is reasonable for cell lines, that the culture cycle time is equal to the doubling time. The second is to base the calculation on a theoretical model for the cell cycle, and we have previously developed such a model, on the basis of the assumption that the transition from G_1_ phase to S phase is controlled by a probability function (Basse *et al*, 2003). Such a model fits experimental data for a number of cell lines treated with paclitaxel and analysed using flow cytometry (Basse *et al*, 2004a, 2004b). The theoretical model provides an equation *T*=−*x*/(log_10_ *P*), where *x* tends upwards towards 4.8, as the G_1_-phase proportion increases towards 100%. Thus, the empirical and theoretical estimates are comparable. There are potential sources of error in this approach, as there are in the *T*_pot_ approach, but the results suggest that the approach is valid.

The results from primary cultures can be compared with the wealth of published data showing a relationship between *T*_pot_ values and survival for several tumour types (Wilson *et al*, 1988; Rew and Wilson, 2000). Although the culture cycle times and *T*_pot_ values are quite different to each other, they both pertain to the cytokinetic properties of tumour cells, and both indicate a surprisingly wide range in culture cycle times. Unfortunately, we have insufficient data linking culture cycle times to more traditional indices of proliferation. Data for Ki-67 staining and mitotic index were available from histology reports of some of the patients, but these were was not correlated with either survival or culture cycle time (results not shown). A larger controlled study would be needed to compare culture cycle times with histological indicators of cell proliferation or with molecular markers, such as cyclin E expression (Keyomarsi *et al*, 2002) and gene expression signatures (Nutt *et al*, 2003). However, one interpretation of the data presented in this study is that cytokinetic properties of tumour cells may be preserved, at least initially, after tumour material is removed from the patient.

The question of why shorter culture cycle times, or shorter *T*_pot_ values, are related to poor clinical outcome still remains to be answered. Such times are not related directly to tumour growth because tumour cells are in a state of continuous turnover, with the net tumour volume doubling time usually in the order of months rather than days (Watson, 1991). Shorter culture cycle times thus reflect higher rates of tumour cell turnover *in vivo*, which in turn might contribute to greater tumour aggressiveness. Rapid turnover of tumour cells may lead to the generation of a more immunosuppressive microenvironment, which could in turn be linked to poor survival (Kim *et al*, 2006).

In conclusion, the overall survival of patients with brain cancers in response to therapy most likely reflects a composite of response of tumour cells and host responses to tumour, and it is clear from this study that tumour cell cytokinetics may play a major role. Other studies have demonstrated that radiotherapy and chemotherapy also contribute to survival, raising the question of whether short-term cultures could provide information on response to therapy as well as on cytokinetics. Some of the patients in this study were treated with radiotherapy (Table 1), and the median survival time for patients receiving radiotherapy (12.1 months) was longer than that for patients not receiving radiotherapy (2.6 months). Although part of this difference will reflect patient selection, much is likely to reflect the contribution of therapy. For 21 of the patients in this study, the response of 7-day cultures to radiotherapy (up to 9 Gy) was also measured (Marshall *et al*, 2003), but no relationship to survival was found (results not shown). Further research will be required to develop accurate methods of assessing the relative contributions of intrinsic tumour cytokinetics and treatment to survival of patients with brain cancer.

## Figures and Tables

**Table 1:** Clinical and biological data

**Patient**	**Age (years)**	**Gender**	**Tumour type**	**Plateau (%)** a	**Culture cycle time (days)**	**Survival (days)** b	**Radiotherapy**
A1	86	M	Glioblastoma	38	9.0	105	Yes
A2	74	M	Metastatic melanoma	75	30.3	559	Yes
B1	54	F	Glioblastoma	41	9.8	271	No
B2	14	F	Glioblastoma	19	5.2	282	Yes
B3	68	F	Glioblastoma	39	9.2	222	Yes
B4	67	F	Glioblastoma	42	10	267	No
B5	57	M	Astrocytoma, anaplastic	28	6.8	101	
B6	52	M	Glioblastoma	89	40.0	489^*^	Yes
C1	73	M	Glioblastoma	78	35.0	399	Yes
D1	32	M	Medulloblastoma	55	14.6	343^*^	Yes
D2	54	M	Glioblastoma	55	14.6	368	Yes
D3	7	F	Astrocytoma, pilocytic	55	14.6	289	No
D4	69	M	Glioblastoma	46	11.2	104	Yes
E1	36	M	Astrocytoma, anaplastic	66	20.9	3168	Yes
E2	49	M	Oligodendroglioma, anaplastic	76	31.7	868^*^	Yes
F1	2	M	Glioblastoma	37	8.8	78	No
F2	72	M	Glioblastoma	26	6.5	197	Yes
F3	74	F	Glioblastoma	38	9.0	491	
F3	68	M	Metastatic melanoma	79	36.9	378	No
G1	43	M	Astrocytoma, anaplastic	64	19.5	316^*^	Yes
G2	13	M	Glioblastoma	59	16.5	476	Yes
G3	35	M	Glioblastoma, recurrent	33	7.9	462	
G2	45	M	Glioblastoma	40	9.5	933	Yes
H1	64	M	Glioblastoma	68	22.6	230	Yes
H2	80	F	Glioblastoma	85	40.0	71	No
H3	73	F	Glioblastoma	36	8.5	38	No
H4	74	M	Astrocytoma, anaplastic	78	35.0	22	
H5	38	F	Medulloblastoma	92	40.0	146	No
H6	40	F	Oligodendroglioma	33	7.9	166^*^	
H5	71	M	Glioblastoma	52	13.3	275	Yes
J1	44	M	Metastatic melanoma	32	7.6	2	No
J2	32	M	Astrocytoma	80	40.0	2023	Yes
J3	58	F	Glioblastoma	50	12.6	380	No
J4	10	F	Medulloblastoma	16	4.7	2195^*^	
K1	76	F	Glioblastoma	56	15.0	21	No
K2	49	M	Metastatic melanoma	38	9.0	251	Yes
K3	47	F	Glioblastoma	33	7.9	156	No
K4	66	M	Glioblastoma	27	6.6	101	No
L1	57	M	Glioblastoma	27	6.6	218	No
L2	69	F	Glioblastoma	16	4.7	46	No
M1	49	M	Glioblastoma	7.3	3.3	111	Yes
M2	43	M	Metastatic melanoma	8.7	3.6	91^*^	
M3	36	F	Medulloblastoma	6.9	3.3	936^*^	Yes
M4	57	M	Metastatic melanoma	11	3.9	18	No
M5	75	M	Astrocytoma, anaplastic	66	20.9	331	Yes
M6	28	M	Astrocytoma, anaplastic	54	14.1	219^*^	No
N1	10	F	Medulloblastoma	63	18.8	455^*^	Yes
N2	42	M	Astrocytoma, anaplastic	49	12.2	1301	Yes
N3	50	M	Glioblastoma	100	40.0	326	Yes
O1	57	F	Glioblastoma	49	12.2	416	Yes
P1	77	F	Glioblastoma	42	10.0	80	No
P2	55	M	Glioblastoma	48	11.9	239	Yes
P3	63	M	Glioblastoma	40	9.5	65	No
R1	65	F	Metastatic melanoma	22	5.7	112	Yes
R2	89	F	Glioblastoma	23	5.9	71	No
R3	73	M	Glioblastoma	17	4.9	71	No
R4	32	F	Glioblastoma	64	19.5	986	Yes
S1	31	F	Oligodendroglioma, anaplastic	55	14.6	2354	Yes
S2	65	F	Glioblastoma	41	9.8	260	Yes
S3	40	M	Metastatic melanoma	1.2	2.0	28	No
S4	53	M	Glioblastoma	3.3	2.6	276	Yes
S5	63	F	Glioblastoma	37	8.8	293	
T1	53	M	Oligoastrocytoma	58	16.0	6464^*^	No
T2	48	F	Glioblastoma	56	15.0	144	Yes
U1	40	F	Glioblastoma	75	30.3	694^*^	Yes
V1	66	F	Glioblastoma	42	10.0	549	Yes
V2	32	F	Glioblastoma	25	6.3	230	Yes
W2	10	M	Medulloblastoma	11	3.9	69	Yes
W3	63	M	Glioblastoma	71	25.4	230	
Y1	69	M	Glioblastoma	86	40.0	47	No

a Results from ^3^H-thymidine incorporation assays, examples of which are shown in Figure 1.

b Patients alive at the time of analysis are marked by an asterisk.

**Table 2:** Associations between survival, culture cycle time and demographic and treatment variables

	**No.**	**Median culture cycle time in days (95% CI)**	**Cycle ⩽10 days, *n* (%)**	**Cycle >10 days, *n* (%)**	**Percentage alive at 1 year (95% CI)** a	**Median survival (95% CI) in months** a
*Age (years)*						
<30	8	11.4 (4.5–17.2)	4 (50%)	4 (50%)	45% (11–75%)	9.5^b^
30–49	22	10.9 (7.6–19.5)	11 (50%)	11 (50%)	62% (37–79%)	30.7 (5.1–66.5)
50–59	12	12.0 (6.7–15.8)	5 (42%)	7 (58%)	42% (15–67%)	9.1 (3.3–13.7)
60–69	14	9.9 (8.8–23.0)	7 (50%)	7 (50%)	14% (2–37%)	7.3 (2.1–8.8)
70+	14	11.7 (8.2–31.0)	7 (50%)	7 (50%)	21% (5–45%)	2.6 (1.2–10.9)
		*P*=0.9	*P*=0.99		*P*=0.002^+^	
						
*Gender*						
Male	41	12.2 (8.9–17.5)	18 (44%)	23 (56%)	39% (24–54%)	8.2 (3.6–12.4)
Female	29	9.8 (8.3–13.3)	16 (55%)	13 (45%)	36% (19–54%)	8.9 (4.8–13.7)
		*P*=0.4	*P*=0.4		*P*=0.9^+^	
						
*Tumour type*						
Glioblastoma	43	9. 8 (9.1–12.6)	23 (53%)	20 (47%)	28% (16–42%)	7.6 (4.7–9.1)
Astrocytoma/oligodendroglioma	13	16.0 (13.0–27.5)	2 (15%)	11 (85%)	64% (30–85%)	66.4 (9.5–104.1)
Medulloblastoma	6	9.9 (3.3–37.8)	3 (50%)	3 (50%)	67%^b^	—c
Metastatic melanoma	8	6.7 (3.1–32.4)	6 (75%)	2 (25%)	31% (5–64%)	3.7 (0.1–12.4)
		*P*=0.06	*P*=0.04		*P*=0.0004^+^	
						
*Radiotherapy*						
Yes	37	13.3 (9.8–18.7)	13 (35%)	24 (65%	51% (34–66%)	12.1 (8.5–18.4)
No	24	9.6 (7.3–14.0)	14 (58%)	10 (42%)	15% (4–33%)	2.6 (1.5–7.2)
Unknown	9	8.1 (4.9–24.2)	7 (78%)	2 (22%)	46% (11–76%)	9.6 (0.7–16.1)
		*P*=0.2	*P*=0.04		*P*=0.0003^+^	

^+^Log rank.

a Calculated using life table methods.

b Numbers too small for calculation.

c Survival curve did not go below 50%.

**Table 3:** Association of culture cycle time, clinical variables and survival estimated using Cox regression

	**Hazard ratio**	**95% CI**	***P*-value**
*Model 1: culture cycle time only*			
*Culture cycle time (days)*			
>10	1.0		
⩽10	2.4	(1.4–4.2)	0.001
			
*Model 2: culture cycle time+age*			
* Culture cycle time (days)*			
>10	1.0		
⩽10	2.5	(1.4–4.4)	0.001
			
* Age group (years)*			
<30	1.0		
30–49	1.0	(0.4–2.9)	0.97
50–59	1.7	(0.6–5.4)	0.33
60–69	3.6	(1.2–10.4)	0.02
70+	3.4	(1.2–9.7)	0.02
			
*Model 3: culture cycle time+age+tumour type*			
* Culture cycle time (days)*			
>10	1.0		
⩽10	2.4	(1.3–4.4)	0.005
			
* Age group (years)*			
<30	1.0		
30–49	0.61	(0.20–1.9)	0.4
50–59	0.86	(0.26–2.8)	0.8
60–69	1.5	(0.47–4.6)	0.5
70+	1.4	(0.46–4.6)	0.5
			
* Tumour type*			
Glioblastoma	1.0		
Medulloblastoma	0.15	(0.03–0.79)	0.03
Metastatic melanoma	1.3	(0.54–2.9)	0.6
Astrocytoma/oligodendroglioma	0.37	(0.13–1.1)	0.07
			
*Model 4: culture cycle time+age+tumour type+radiotherapy (days)*			
* Culture cycle time*			
>10	1.0		
⩽10	2.9	(1.5–5.9)	0.003
			
* Age group (years)*			
<30	1.0		
30–49	0.74	(0.25–2.2)	0.6
50–59	0.53	(0.16–1.7)	0.3
60–69	1.4	(0.47–4.5)	0.5
70+	1.8	(0.60–5.6)	0.3
			
* Tumour type*			
Glioblastoma	1.0		
Medulloblastoma	0.21	(0.04–1.0)	0.05
Metastatic melanoma	0.93	(0.38–2.3)	0.9
Astrocytoma/oligodendroglioma	0.30	(0.09–0.92)	0.03
			
* Radiotherapy*			
No	1.0		
Yes	0.32	(0.16–0.63)	0.001
Unknown	0.14	(0.05–0.43)	0.001
